# Angiotensin-Converting Enzyme 2 in the Pathogenesis of Renal Abnormalities Observed in COVID-19 Patients

**DOI:** 10.3389/fphys.2021.700220

**Published:** 2021-08-23

**Authors:** Nayara Azinheira Nobrega Cruz, Lilian Caroline Gonçalves de Oliveira, Helio Tedesco Silva Junior, Jose Osmar Medina Pestana, Dulce Elena Casarini

**Affiliations:** Nephrology Division, Department of Medicine, Escola Paulista de Medicina, Universidade Federal de São Paulo, São Paulo, Brazil

**Keywords:** angiotensin-converting enzyme 2, coronavirus disease 2019, severe acute respiratory syndrome coronavirus 2, acute kidney injury, renin-angiotensin system, kallikrein-kinin system

## Abstract

Coronavirus disease 2019 (COVID-19) was first reported in late December 2019 in Wuhan, China. The etiological agent of this disease is severe acute respiratory syndrome coronavirus 2 (SARS-CoV-2), and the high transmissibility of the virus led to its rapid global spread and a major pandemic (ongoing at the time of writing this review). The clinical manifestations of COVID-19 can vary widely from non-evident or minor symptoms to severe acute respiratory syndrome and multi-organ damage, causing death. Acute kidney injury (AKI) has been recognized as a common complication of COVID-19 and in many cases, kidney replacement therapy (KRT) is required. The presence of kidney abnormalities on hospital admission and the development of AKI are related to a more severe presentation of COVID-19 with higher mortality rate. The high transmissibility and the broad spectrum of clinical manifestations of COVID-19 are in part due to the high affinity of SARS-CoV-2 for its receptor, angiotensin (Ang)-converting enzyme 2 (ACE2), which is widely expressed in human organs and is especially abundant in the kidneys. A debate on the role of ACE2 in the infectivity and pathogenesis of COVID-19 has emerged: Does the high expression of ACE2 promotes higher infectivity and more severe clinical manifestations or does the interaction of SARS-CoV-2 with ACE2 reduce the bioavailability of the enzyme, depleting its biological activity, which is closely related to two important physiological systems, the renin-angiotensin system (RAS) and the kallikrein-kinin system (KKS), thereby further contributing to pathogenesis. In this review, we discuss the dual role of ACE2 in the infectivity and pathogenesis of COVID-19, highlighting the effects of COVID-19-induced ACE2 depletion in the renal physiology and how it may lead to kidney injury. The ACE2 downstream regulation of KKS, that usually receives less attention, is discussed. Also, a detailed discussion on how the triad of symptoms (respiratory, inflammatory, and coagulation symptoms) of COVID-19 can indirectly promote renal injury is primary aborded.

## Introduction

Severe acute respiratory syndrome coronavirus 2 (SARS-CoV-2) is responsible for the outgoing pandemic of coronavirus disease 2019 (COVID-19; Liu et al., 2020; Zhang and Holmes, 2020). Acute kidney disease is a complication of COVID-19; however, data on the percentage of acute kidney injury (AKI) among hospitalized COVID-19 patients are conflicting, varying from 6% in early reports to 20–36% in more recent researches (Cheng et al., 2020; Hirsch et al., 2020; Xiao et al., 2020). The incidence of AKI in COVID-19 patients is significant, and there is consistent evidence of its association with disease severity and mortality (Cheng et al., 2020; Diao et al., 2020; Hirsch et al., 2020).

The high affinity of SARS-CoV-2 for its receptor, angiotensin (Ang)-converting enzyme 2 (ACE2), may play a significant role in tissue tropism as ACE2 is widely distributed in human organs. ACE2 is particularly abundant in kidneys and can be involved in the mechanisms leading to kidney injury in COVID-19 (Hamming et al., 2004; Diao et al., 2020; Hoffmann et al., 2020).

**Figure 1:**
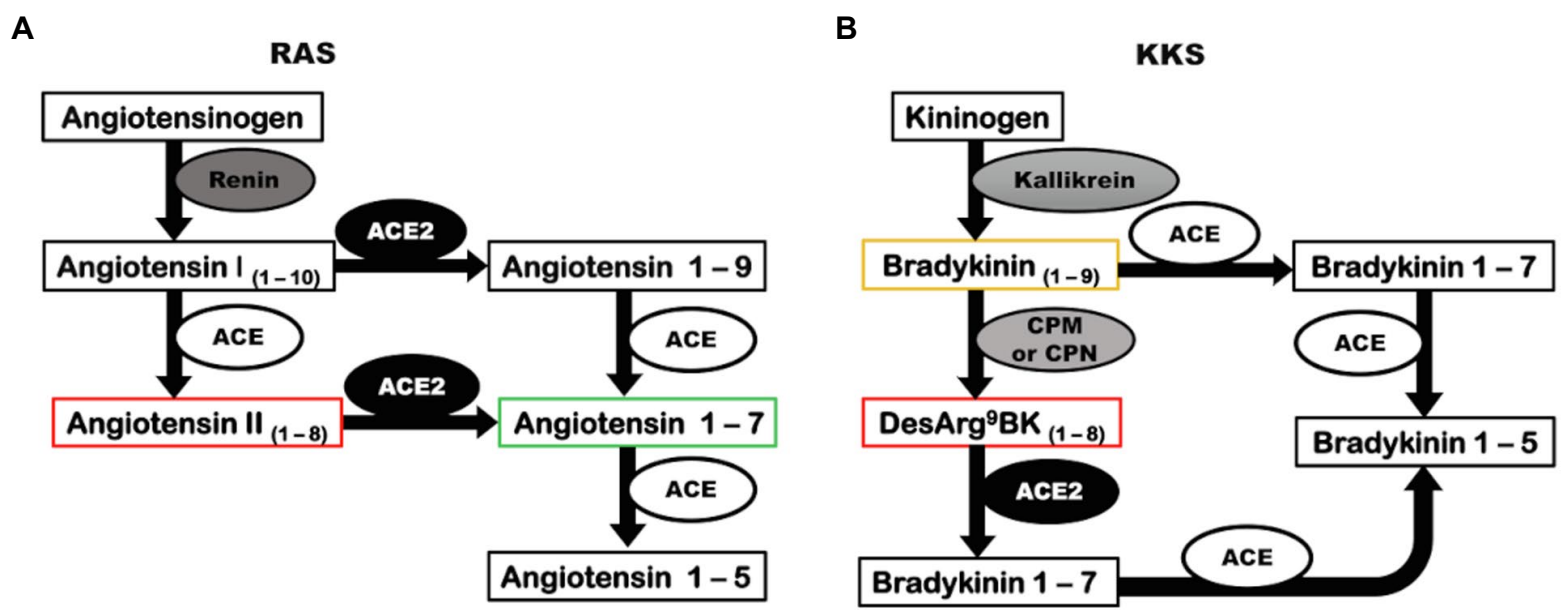
Angiotensin-converting enzyme 2 has a catalytic role in RAS and KKS. **(A)** Renin converts the precursor, angiotensinogen, into angiotensin I. In a classic pathway, angiotensin I is cleaved by ACE to form Angiotensin II. ACE2 can biosynthesize angiotensin 1–7 by two distinct pathways: acting directly on angiotensin II or alternatively converting angiotensin I into angiotensin 1–9 that is further cleaved by ACE, generating angiotensin 1–7. **(B)** The precursor kininogen is cleaved by kallikrein to form the active peptide, bradykinin that is rapidly degraded by ACE, or in an alternative pathway, can be converted to desArg^9^bradykinin by CPM and CPN. ACE2 can inactivate desArg^9^bradykinin. ACE, angiotensin-converting enzyme; ACE2, angiotensin-converting enzyme 2; CPM, carboxypeptidase M; CPN, carboxypeptidase N; KKS, kallikrein-kinin system; and RAS, renin-angiotensin system.

Angiotensin-converting enzyme 2 was discovered in 2000 by two distinct research groups (Donoghue et al., 2000; Tipnis et al., 2000). ACE2 is a zinc metallopeptidase that releases a single amino acid from the carboxy-terminal of its substrates (Guang et al., 2012) and integrates two important physiological systems: the renin-angiotensin system (RAS) and kallikrein-kinin system (KKS; Figure 1; Vickers et al., 2002; Guang et al., 2012). In RAS, ACE2 cleaves the Ang II to form Ang 1–7 (Figure 1A; Vickers et al., 2002). Ang II is a peptide that has vasoconstrictor, anti-natriuretic, anti-diuretic, inflammatory, oxidant, and fibrotic effects through its receptor of type 1 (AT1), and is known to be elevated in hypertension, kidney diseases, and metabolic disorders (Chappell, 2016; Muñoz-Durango et al., 2016). Ang 1–7 has opposite actions, exerting vasodilation, natriuresis, diuresis, anti-inflammatory, antioxidant, and anti-fibrotic actions binding to its receptor, Mas (Santos, 2014). In addition, ACE2 can act on Ang I to release Ang 1–9, which is further converted to Ang 1–7 by angiotensin-converting enzyme (ACE; Figure 1A). However, ACE2 has a higher catalytic efficiency for the hydrolysis of Ang II than Ang I (Vickers et al., 2002). Thus, the main biological function of ACE2 is to counterbalance the deleterious effects of the ACE/Ang II/AT1 axis of RAS (Santos, 2014).

In KKS, ACE2 cannot degrade bradykinin (BK), the main kinin of the system that exerts vasodilatory, natriuretic, diuretic, and inflammatory effects, and interacts with the constitutive kinin receptor type 2 (B2); however, ACE2 inactivates desArg^9^bradykinin (desArg^9^BK), a kinin intimately involved in inflammatory pathologies that exerts its effects by binding to the inducible kinin receptor B1 (Figure 1B; Kakoki and Smithies, 2009; Klein et al., 2010; Guang et al., 2012; Zhang et al., 2018). The ability of ACE2 to cleave desArg^9^BK and Ang II, both involved in inflammatory processes, suggests that the ACE2/Ang 1–7/Mas axis is an important anti-inflammatory pathway.

The identification of ACE2 as the receptor of SARS-CoV-2 has prompted a debate on how the ACE2 can influence the course of the COVID-19 (Hoffmann et al., 2020; Lanza et al., 2020; Verdecchia et al., 2020). Due to wide distribution of ACE2 in humans, the higher expression of this enzyme may enhance infectivity (Pinto et al., 2020). However, the depletion of the biological functions of ACE2 due to the internalization of the receptor along with SARS-CoV-2, leads to impairment of RAS and KKS, which can contribute to COVID-19 pathogenesis (Lanza et al., 2020; Verdecchia et al., 2020).

In this context, the kidneys are a potential target for SARS-CoV-2, as podocytes and proximal tubule cells abundantly express ACE2, and their role in urine filtration allows contact with circulating viruses (Hamming et al., 2004; Cheng et al., 2020; Pan et al., 2020). In addition, the kidneys are particularly sensitive to ACE2 downregulation, which is associated with several kidney diseases (Mizuiri and Ohashi, 2015). Elucidating the mechanisms responsible for renal involvement in COVID-19 and determining the immediate and long-term impacts on kidney function are necessary for achieving better patient management and developing therapeutic strategies to eliminate or minimize kidney damage.

## Sars-Cov-2 and Covid-19 Background

Three major outbreaks have been caused by severe acute respiratory syndrome coronaviruses (SARS-CoVs) in the last 2 decades: severe acute respiratory syndrome (SARS-CoV) in 2002, Middle East respiratory syndrome (MERS-CoV) in 2012, and the outgoing COVID-19 in 2019 caused by the novel coronavirus named, SARS-CoV-2. There is great epidemiological concern regarding these viral agents due to their transmissibility and mortality (Liu et al., 2020; Zhang and Holmes, 2020).

The new coronavirus, SARS-CoV-2, was identified in late December 2019 in Wuhan, China (Zhu et al., 2020). As of July 2021, the COVID-19 pandemic is still outgoing and has affected more than 189,000,000 people and caused more than 4,000,000 deaths worldwide (Johns Hopkins University, 2021a). Currently, no treatment has proven to be safe and efficient, despite the significant number of clinical trials to repurpose approved drugs or to develop new drugs specific for COVID-19 treatment (Duan et al., 2020; Senger et al., 2020). Recently, different vaccines have been approved for emergency use to combat COVID-19 by federal agencies: the BioNTech-Pfizer, Moderna, and Janssen vaccines were approved by the FDA in North America. In Brazil, for example, CoronaVac, AstraZeneca, and Janssen vaccines were approved for emergency use. More than 3 billion doses of vaccines against SARS-CoV-2 were administered worldwide (Johns Hopkins University, 2021a).

### SARS-CoV-2 Structure and Cell Entry Mechanism

**Figure 2:**
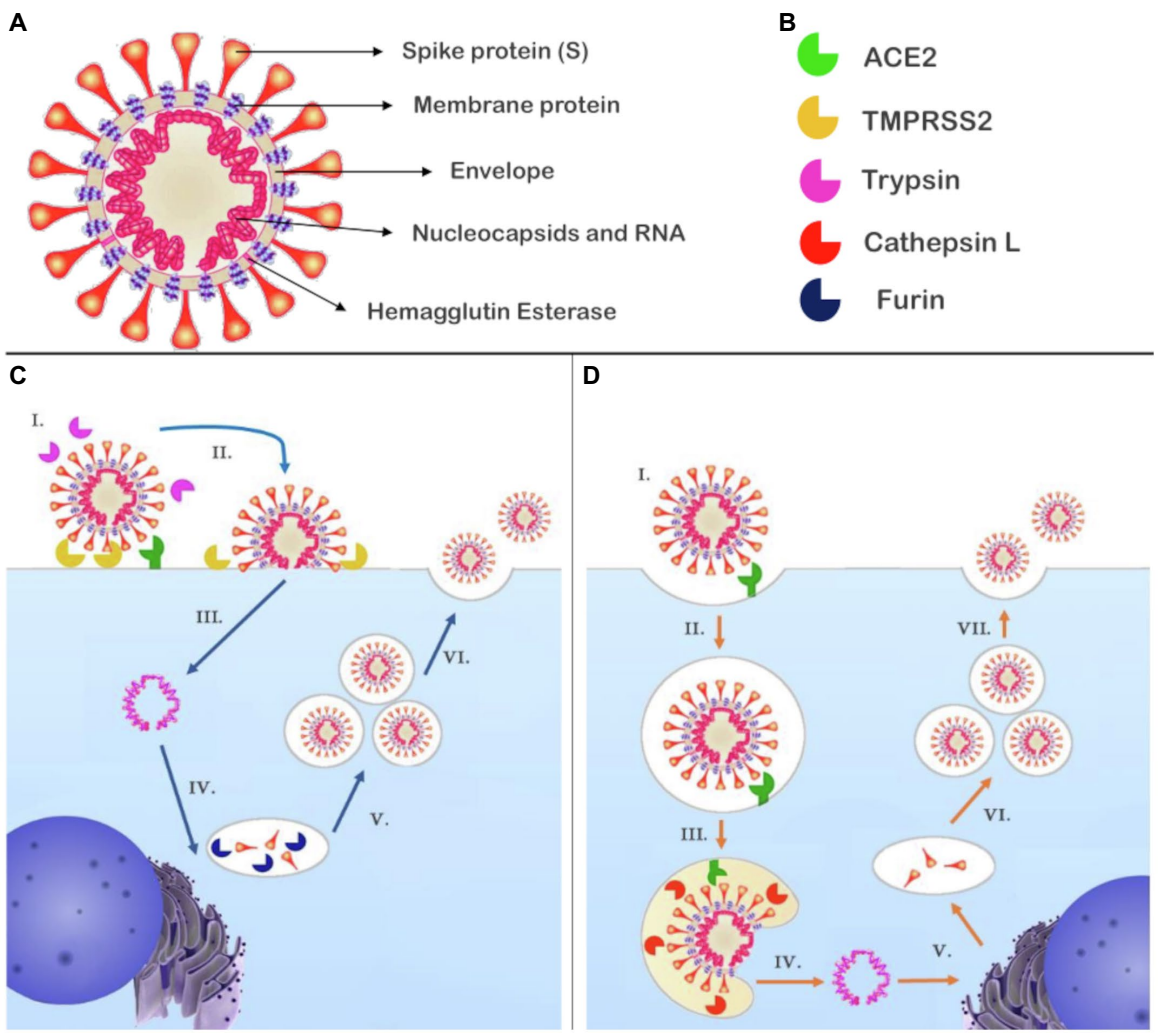
Structure of SARS-CoV-2 and the proposed mechanisms of cell entry and replication. **(A)** SARS-CoV-2 is composed of RNA contained in nucleocapsids; the envelope retains the genetic material and contains the membrane protein and hemagglutinin esterase dimers. Attached to the envelope is the spike protein (S) responsible for receptor binding. **(B)** Enzymes reported to participate in the cell entry mechanism of SARS-CoV-2. **(C)** (I.) SARS-CoV-2 recognizes its receptor, ACE2. The short pathway is possible if S had been primed by furin during biosynthesis and in the presence of TMPRSS2 and/trypsin that cleave S1; (II.) the virus fuses with plasmatic membrane; (III.) releasing its genetic material into the cytosol; (IV.) RNA transcription and replication occur in the cytosol, while the structural proteins are biosynthesized in the endoplasmic reticulum and Golgi apparatus, at this point, furin can prime S at S1/S2; and (V.) New genetic material is encapsulated by envelope and structural proteins generating new virions (VI.) that will be released from the host cell. **(D)** (I.) The SARS-CoV-2 recognizes its receptor, ACE2. If S had not been primed, a second pathway is activated and (II.) the virus is endocytosed; (III.) Owing to the decreased pH, cathepsin L can be activated and cleaves S1, promoting fusion of the SARS-CoV-2 with the endosome membrane and (IV.) the release of viral genetic material; (V.) RNA transcription and replication occur in the cytosol while biosynthesis of the structural proteins occurs in the endoplasmic reticulum and Golgi apparatus. In this representation, there is no furin to prime S at S1/S2; and (VI.) The genetic material is encapsulated by envelope and structural proteins, generating new virions (VII.) that will be released from the host cell. ACE2, angiotensin-converting enzyme 2; S, spike protein; SARS-CoV-2, severe acute respiratory syndrome coronavirus 2; and TMPRSS2, transmembrane serine protease 2.

Severe acute respiratory syndrome coronavirus 2 is a single positive-strand RNA virus belonging to the betacoronavirus B lineage. Structurally, SARS-CoV-2 is comprised of a S spike protein (S), a membrane protein, an envelope protein, nucleocapsids, hemagglutinin-esterase dimers, and its genetic material (Figure 2A; Walls et al., 2020).

The S protein is a transmembrane glycoprotein that can be divided into S1 and S2 subunits. The S1 subunit contains the receptor binding domain (RBD), which is the most variable part of the coronavirus genome and is responsible for the high affinity of SARS-CoV-2 for human ACE2, which acts as a receptor for virus internalization (Hoffmann et al., 2020; Wang et al., 2020). The RBD has a dynamic position; in SARS-CoV-2, it is found predominantly lying down, which allows the virus to evade the immune system; however, the RBD only interacts with ACE2 when standing up; once this conformation is less frequent, the higher affinity may be the result of an adaptive change (Shang et al., 2020).

Proteolytic activation of S is a crucial step for membrane fusion; the process promotes conformational changes that release sufficient energy to overcome the lipid bilayer fusion energy barrier (Millet and Whittaker, 2015). SARS-CoV-2 fusion mechanisms have been proposed based on current evidence and previous studies of MERS-CoV and SARS-CoV. To catalyze the fusion process, the SARS-CoV-2 S unit should be preactivated (priming) by proteolytic proteases; there are two cleavage points in S, the first is found between S1 and S2 and is a polybasic furin cleavage site while the second is found in the S2 sequence that can be cleaved by multiple proteases, including trypsin, cathepsin L, and transmembrane serine protease 2 (TMPRSS2; Figure 2B; Hoffmann et al., 2020; Shang et al., 2020; Walls et al., 2020).

There are two possible pathways for the fusion. The plasma membrane route is possible if exogenous or transmembrane proteases, such as trypsin and TMPRSS2, are present. In MERS-CoV, this pathway only occurs if S is cleaved by furin-like proteases at the link between S1 and S2 subunits during biosynthesis (Figure 2C; Tang et al., 2020). Otherwise, S1/S2 are cleaved after S binds to ACE2, activating a second entry pathway where the virus is endocytosed (Figure 2D). Within the endosome, cathepsin L can be activated by low pH and cleaves S at the S2 cleavage site, triggering fusion of the virus with the endosomal membrane (Shulla et al., 2011; Tang et al., 2020). Independent of the pathway by which the viral genome reaches the cytosol, copies of the virus genome are transcribed in the cytoplasm, and structural proteins are synthetized in the intermediate compartment between the endoplasmic reticulum and Golgi apparatus, which allows S to be cleaved by furin during its biosynthesis depending on the host cells (Tang et al., 2020; Walls et al., 2020). In fact, the presence of TMPRSS2 has been reported as a limiting factor for SARS-CoV-2 cell entry. Further, the presence of the polybasic furin cleavage site in S, wide distribution of furin-like proteases, and cathepsin L in humans are features, which contribute to enhance virus fusion (Hoffmann et al., 2020; Walls et al., 2020).

Tissue tropism is influenced by multiple factors, with receptor expression, distribution, and attachment to receptors as fundamental aspects to ensure that the virus entry a variety of host’s cells, targeting different organs (Maginnis, 2018). The high affinity of SARS-CoV-2 RBD for ACE2 in conjunction with the wide distribution of the ACE2 and colocalization with TMPRSS2, which allows S2 subunit release and fusion to host cells, may imply the broad clinical manifestations of COVID-19, ranging from subclinical symptoms to severe acute respiratory syndrome and multiple organ damages (Shulla et al., 2011; Hoffmann et al., 2020; Walls et al., 2020; Yang et al., 2020). The presence of a polybasic furin cleavage site in the S protein of SARS-CoV-2 also expands tropism because furin-like proteases are near-ubiquitous and distributed in human cells (Walls et al., 2020).

### Renal Abnormalities Related to COVID-19

In relation to the clinical manifestation of COVID-19, 30% of the cases can be asymptomatic; most of the symptomatic cases (approximately 86%) are characterized by mild to moderate symptoms (Li et al., 2020b; Yang et al., 2020). However, in 14% of the symptomatic cases, more severe symptoms are present, and hospitalization and oxygen therapy are required (Li et al., 2020b; Yang et al., 2020). Multiple organ damage is likely to be a complication among severely ill patients. The lungs are the most affected organ, but AKI, liver dysfunction, and cardiac injury are also commonly seen (Guo et al., 2020; Yang et al., 2020). The global case fatality rate of COVID-19 is estimated to be approximately 2.2% (Johns Hopkins University, 2021b). However, among critically ill patients, the rate increases to 61.5%. Mortality risk is associated with age, presence of underlying diseases, and development of organ damage during COVID-19 (Li et al., 2020b; Yang et al., 2020).

Abnormalities related to impaired kidney function are commonly seen upon admission of COVID-19 patients, with elevated serum creatinine and blood serum urea present in 14.4 and 13% of patients, respectively (Cheng et al., 2020; Hirsch et al., 2020). A reduced glomerular filtration rate (GFR) has also been observed in 13.1% of the COVID-19 patients on admission (Cheng et al., 2020). Proteinuria and hematuria are relatively common, affecting 28–43.9% and 19–26.7%, respectively. These patients were predominantly males and elderly people (Chaudhri et al., 2020; Cheng et al., 2020; Nadim et al., 2020). The high incidence of proteinuria and hematuria among COVID-19 patients raises concern because proteinuria is associated with the development of AKI and higher mortality (Chaudhri et al., 2020; Cheng et al., 2020), and patients with hematuria on admission are more prone to intensive care unit (ICU) admission, invasive mechanical ventilation, and death (Chaudhri et al., 2020).

The first reports described that AKI affects only 5–6% of total COVID-19 in-hospital patients (Cheng et al., 2020); however, in more recent studies, the reported incidence of AKI has ranged from 19 to 57% (Chan et al., 2020; Hirsch et al., 2020; Xiao et al., 2020; Nugent et al., 2021). These differences may be related to the main target population and to the different SARS-CoV-2 variants. At the beginning of the spread of COVID-19, the disease was primarily concentrated in Asia and, currently, occidental countries are the epicenter of COVID-19. This shift may impact the severity of COVID-19 and the incidence of AKI that increased mainly among patients admitted to the ICU, where COVID-19-associated AKI affects over to 60% of patients (Diao et al., 2020; Dudoignon et al., 2020).

The incidence of AKI as an outcome of COVID-19 is relatively high; 19% of patients require kidney replacement therapy (KRT) and the presence of AKI is a risk factor for mortality (Chawla et al., 2017; Chan et al., 2020). The reported mortality rate among patients who develop AKI is conflicting. A prospective cohort study with 701 COVID-19 patients from a hospital in Wuhan, China reported that the mortality rate reached 91.7% among those who developed AKI (Cheng et al., 2020). In a more recent study with 5,449 patients from hospitals within the metropolitan region of New York, AKI was present in 1,993 patients. Among these patients with AKI, 519 (26%) were discharged, 698 (35%) died, and 777 (39%) remained hospitalized at the time of publication (Hirsch et al., 2020). There are limited reports on renal recovery in survivors of COVID-19-associated AKI. The actual reported rate of full recovery at discharge and during post-hospital follow-up ranges from 65 to 82.4% (Chan et al., 2020; Nugent et al., 2021; Stockmann et al., 2021). However, a comparison between COVID-19-related AKI and general AKI after adjustments shows that GFR declines faster in patients with COVID-19-related AKI; these patients are also more likely to require KRT than patients with AKI who tested negative to COVID-19. Further, recovery is slower, and the full recovery rate is lower in patients with COVID-19-associated AKI (Nugent et al., 2021).

## Dual Role of Ace2 in Sars-Cov-2 Infectivity and Pathogenesis

Angiotensin-converting enzyme 2 is specifically recognized by its actions counterbalancing the deleterious effects of the ACE/Ang II/AT1 axis of RAS. However, the enzyme also participates in KKS, which is responsible for inactivating desArg^9^BK and has functions beyond its catalytic actions. Additionally, ACE2 plays a role in the transport of amino acids in the kidneys and intestine, and ACE2 participates in pancreatic insulin secretion (Guang et al., 2012; Hashimoto et al., 2012; Santos et al., 2019).

The discovery of ACE2 as the receptor of SARS-CoV and now SARS-CoV-2 has led to a debate regarding the role of ACE2 in COVID-19: (i) whether ACE2 upregulation enhances SARS-CoV-2 infectivity and can be related to more severe cases and (ii) whether SARS-CoV-2 binding reduces ACE2 bioavailability, which causes impairment of RAS that is associated with a more severe disease.

### Upregulation of ACE2 May Enhance SARS-CoV-2 Infectivity

In SARS-CoV infection, it has been reported that the overexpression of ACE2 enhanced viral entry into the cells and mice treatment with the anti-ACE2 antibodies ceased viral entry. In addition, ACE2 knockout mice have milder SARS-CoV outcomes than wild-type animals (Verdecchia et al., 2020).

A systematic review of lung transcriptome analysis comparing healthy non-smokers with smokers, chronic obstructive pulmonary disease (COPD), and pulmonary arterial hypertension volunteers, revealed an increase in ACE2 expression in patients with lung diseases that are more likely to develop severe COVID-19 (Pinto et al., 2020). It is worth noting that increased expression of the *ACE2* gene in lung disease is associated with an increase in ADAM-10 expression, which sheds ACE2 in the pulmonary epithelium (Pinto et al., 2020).

A similar study did not find a difference between COPD patients and healthy patients but showed that smoke causes an acute increase in ACE2 expression and SARS-CoV promotes ACE2 expression in infected cells (Li et al., 2020a). However, gene and actual protein expression in the tissue are not always correlated. In lipopolysaccharide (LPS)-induced lung injury, ACE2 protein expression and activity are decreased, despite a rapid increase in its mRNA expression. The disparity between protein levels and mRNA can imply a feedback response or post-transcriptional modulation of ACE2 by LPS (Sodhi et al., 2018). Local attenuation of ACE2 functions due to shedding and post-transcriptional internalization after LPS stimulation has been reported (Sodhi et al., 2018).

### SARS-CoV-2 May Reduce ACE2 Bioavailability and Downregulate Its Biological Functions

**Figure 3:**
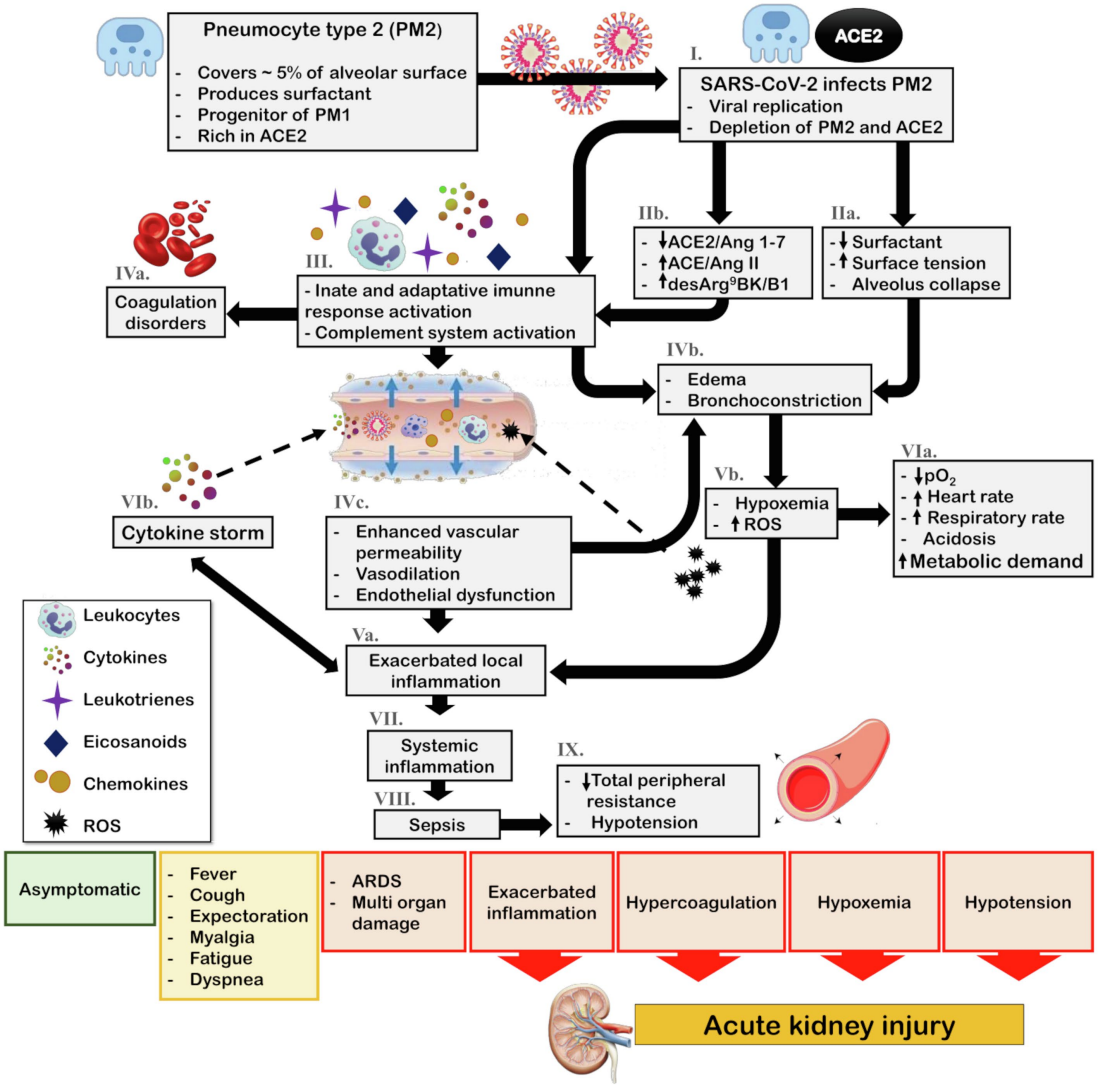
Schematic representation of COVID-19 pathophysiology and its indirect effects on kidney. The main target of SARS-CoV-2 in the lungs is PM2. (I.) Viral infection and replication can culminate the depletion of PM2 and ACE2 reservoir. Consequently, (IIa.) surfactant production diminishes, leading to an increase in surface tension and alveolar collapse. Additionally, (IIb.) SARS-CoV-2 infection associated with ACE2/Ang 1–7 downregulation and ACE/Ang II and desArg^9^BK/B1 exacerbation promotes the (III.) activation of the innate and adaptative immune response and complement system leading to recruitment of leukocytes and release of cytokines, chemokines, eicosanoids, and leukotrienes. The complement system and eicosanoids promote (IVa.) coagulation disorders and the leukotrienes in association with increased surface tension contribute to (IVb.) bronchoconstriction. Moreover, the local inflammation culminates in (IVc.) increased vascular permeability, vasodilation, and endothelial dysfunction, thereby enhancing leukocyte recruitment, and leading to (Va.) exacerbated local inflammation what also contribute to (IVb.) bronchoconstriction and edema. Furthermore, bronchoconstriction and edema lead to (Vb.) hypoxia and ROS generation, which contributes to (VIa.) cardiorespiratory alterations that increase metabolic demand. Most importantly, ROS generation feeds the cycle of (Va.) enhanced inflammation. This inflammatory state leads to the (VIb.) cytokine storm and due to (IVc.) enhanced vascular permeability, viral particles, leukocytes, ROS, and cytokines can reach the blood stream, ultimately causing (VII.) systemic inflammation, (VIII.) sepsis, and consequently (IX.) hypotension. Depending on the patient health status, the steps in the pathophysiological cascade that are activated, and the intensity of the immune response, the clinical manifestations can vary from asymptomatic and mild symptoms, such as fever, cough, and myalgia, to severe symptoms, including acute respiratory distress and multi-organ damage. In this scenario, exacerbated inflammation, coagulation disorders, hypoxemia, and hypotension contribute to acute kidney injury (AKI). ACE, angiotensin-converting enzyme; ACE2, angiotensin-converting enzyme 2; Ang 1–7, angiotensin 1–7; Ang II, angiotensin II; B1, kinin receptor type 1; COVID-19, coronavirus disease 2019; desArg^9^BK, desArg^9^bradykinin; PM1, pneumocytes type I; PM2, pneumocytes type II; ROS, reactive oxygen species; and SARS-CoV-2, severe acute respiratory syndrome coronavirus 2.

In the lungs, ACE2 is mainly expressed in pneumocyte type 2 (PM2) and macrophages. The PM2 are crucial cells for lung function as they are responsible for producing alveolar surfactant and are the progenitor cells for type 1 pneumocytes that perform gas exchanges and comprise 95% of the pneumocyte cells (Verdecchia et al., 2020). The binding and fusion of SARS-CoV and SARS-CoV-2 to cells induce a reduction in bioavailability of the ACE2 receptor, which is internalized with the virus (Figure 3; Kuba et al., 2005; Verdecchia et al., 2020). Depletion of ACE2 promotes the imbalance of local RAS with an increase in the Ang II/Ang 1–7 ratio, which promotes proinflammatory responses.

Briefly, Ang 1–7 diminishes macrophage and lymphocyte infiltration and regulates the ERK1/2 pathway, which induces the production of interleukin (IL)-10, an anti-inflammatory and pro-resolutive cytokine (Mahmudpour et al., 2020). In addition, Mas activation by Ang 1–7 inhibits AT1 (Roks et al., 1999; Clark et al., 2001). Conversely, the Ang II acts *via* AT1 to activate the complement cascade (C5a and C5b-9). Furthermore, activation of the innate immunity is closely related to coagulation. Coagulation disorders are a common feature in severely ill patients with COVID-19 (Liao et al., 2020). In addition, Ang II stimulates the nuclear factor kappa B (NFκB) pathway, leading to the production of cytokines [IL-6, tumor necrosis factor (TNF)-α, IL-1B, and IL-10] and regulates mitogen-activated protein kinases (MAPK), which play important roles in the release of cytokines (IL-1, IL-10, IL-12, and TNF-α; Lu et al., 2020; Mahmudpour et al., 2020). SARS-CoV-2 and Ang II have a synergistic effect on the activation of the NFκB machinery, known as IL-6 amplifier. This is an important step in the cytokine storm, a major aggravating of COVID-19 which is associated with higher mortality (Figure 3; Lu et al., 2020; Mahmudpour et al., 2020).

Although most of the biological functions of ACE2 are related to Ang II and Ang 1–7 balance, desArg^9^BK plays an important role in inflammatory processes and is modulated by ACE2 (Sodhi et al., 2018). Interestingly, IL-1B and TNF-α can induce B1 expression. B1 activation promotes the release of chemokines, increases the expression of IL-1B and monocyte chemoattractant protein 1 (MCP-1), and enhances the recruitment and infiltration of neutrophils (Figure 3; Mahmudpour et al., 2020).

Several experimental and clinical models of lung inflammation have reported beneficial roles of ACE2/Ang 1–7/Mas, including reduced infiltration of lymphocytes and neutrophils, reduction of perivascular and peri-bronchiolar inflammation, and decreased production of IL-6 and TNF-α (Verdecchia et al., 2020). In an acid aspiration experimental model of acute lung injury, the ACE2 knockout animals had more severe inflammatory lesions that were attenuated with recombinant ACE2 and administration of the angiotensin II receptor blocker (ARB; Sodhi et al., 2018; Verdecchia et al., 2020). The isolated S spike of SARS-CoV could induce ACE2 downregulation with concomitant increase in Ang II levels. Further, ARBs were found to reduce severe inflammatory pulmonary lesions (Kuba et al., 2005). In LPS-induced lung injury, ACE2 activity was reduced. Further, its activity promoted the accumulation of desArg^9^BK and overexpression of B1, and enhanced neutrophil recruitment and infiltration (Sodhi et al., 2018). In addition, higher circulatory levels of Ang II were observed in COVID-19 patients compared to control subjects, and the levels of Ang II were found to correlate with lung injury (Mahmudpour et al., 2020).

Additionally, the groups at risk of developing more severe COVID-19 and have high mortality rates include older adults, males, and people with chronic diseases, including diabetes, hypertension, and cardiovascular and kidney diseases (Williamson et al., 2020; Yang et al., 2020). Interestingly, in all these groups, impairment of RAS with reduced ACE2/Ang 1–7/Mas regulation and/or enhanced ACE/Ang II/AT1 actions has been reported. Therefore, the depletion of ACE2 due to SARS-CoV-2 infection can contribute at least in part to the triad of hematological, pulmonary, and inflammatory outcomes of COVID-19 (Lanza et al., 2020; Verdecchia et al., 2020).

## Possible Mechanisms Involved in Kidney Injury in Covid-19 Patients

**Figure 4:**
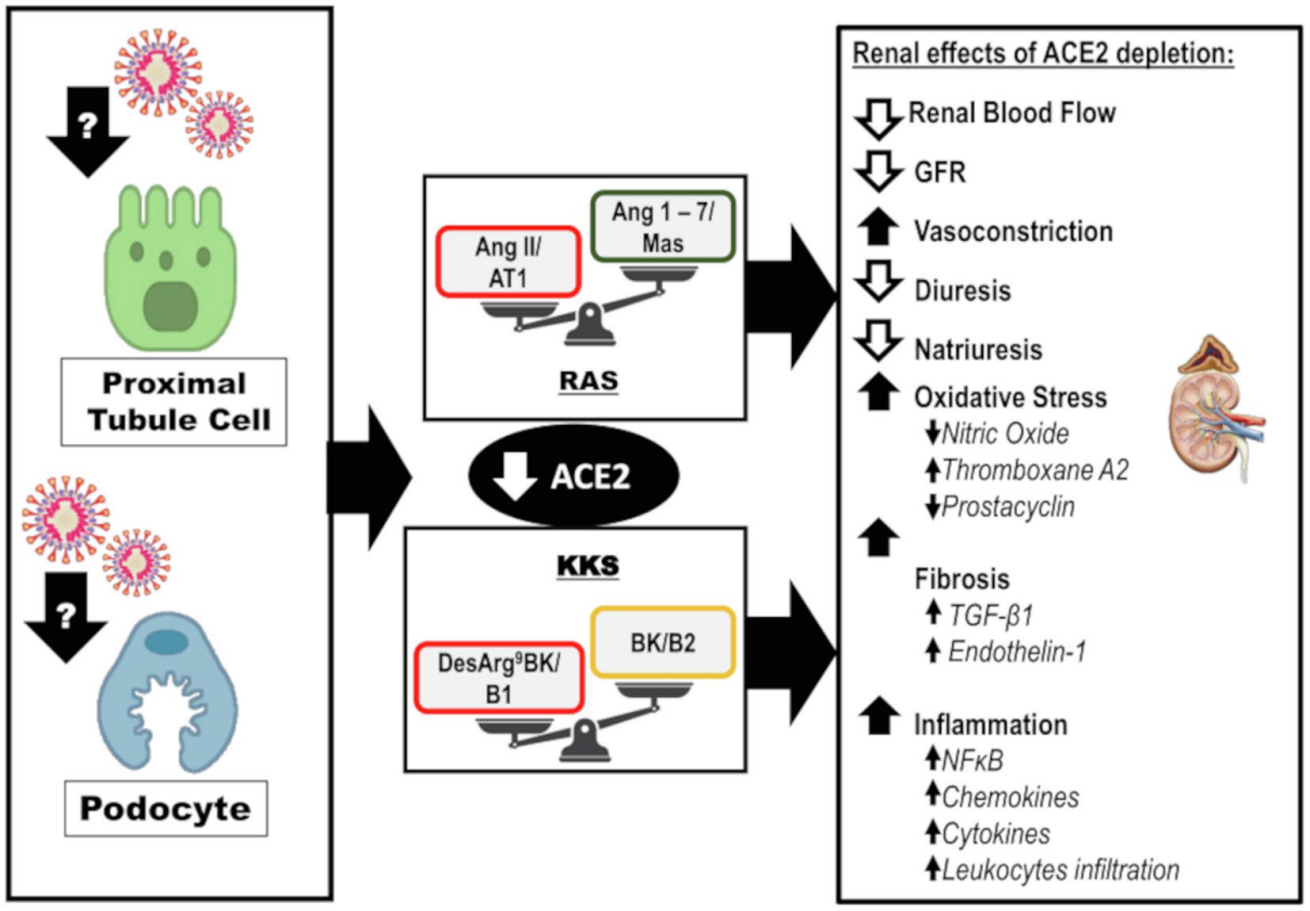
Direct effects of SARS-CoV-2 on kidneys: Infection and disruption of the downstream mechanisms regulated by ACE2. It is likely that SARS-CoV-2 infects the kidney by directly targeting the proximal tubule cells and podocytes. The infection can lead to depletion of ACE2 and its biological functions at the intrarenal level, which may lead to exacerbated actions of the ACE/Ang II/AT1 axis of RAS and desArg^9^BK/B1 axis of KKS. This impairment leads to reduced renal blood flow, GFR, diuresis, and natriuresis. However, there is an increase in vasoconstriction. The oxidative stress is enhanced due to a decrease in NO levels, interfering in the balance between prostacyclin and thromboxane A2. Furthermore, fibrosis is enhanced in response to TGF-β1 and endothelin-1. Finally, inflammation is upregulated along with augmented levels of chemokines, cytokines, and leukocyte recruitment. ACE2, angiotensin-converting enzyme 2; AT1, angiotensin II receptor type 1; BK, bradykinin; desArg^9^BK, desArg^9^bradykinin; B1, kinin receptor type 1; B2, kinin receptor type 2; GFR, glomerular filtration rate; KKS, kallikrein-kinin system; and RAS, renin-angiotensin system.

The exact mechanism of kidney involvement in COVID-19 is unknown and might be multifactorial. Indirect injury due to systemic inflammation, hypoxemia, shock, hypotension, and systemic imbalance of RAS associated with SARS-CoV-2 infection is possible (Figure 3). Additionally, the SARS-CoV-2 can infect renal cells causing direct injury and subsequent impairment of intrarenal RAS that may be a major contributor to acute and long-term kidney injury (Figure 4).

### SARS-CoV-2 Indirect Effects on Kidneys: How the Inflammatory, Coagulation, and Respiratory Symptoms Can Lead to Kidney Injury

Acute kidney injury is associated with intrarenal and systemic inflammatory responses (Rabb et al., 2016). After an insult, morphological and metabolic alterations occur in tubular epithelium and endothelial cells, inducing the synthesis and release of cytokines, chemokines, and leukocyte infiltration (Akcay et al., 2009). Inflammation plays a major role in the onset and progression of AKI (Akcay et al., 2009; Rabb et al., 2016). In fact, there is a cytokine profile associated with AKI, and IL-18 and IL-6 are considered biomarkers for AKI (Akcay et al., 2009).

After SARS-CoV-2 enters the cells and replicates, especially in type II pneumocytes in the lungs, innate and adaptive immune responses are activated (Miyazawa, 2020; Figure 3). Complement activation is an important feature of the innate immune response, which is the primary defense line to be activated after infection (Kenawy et al., 2015). Complement activation is strongly influenced by a pH below 7.1; inflammation decreases pH locally, allowing its activation. In addition, some fluid compartments, such as the lumen of renal tubules, can naturally present a pH lower than 7.1 (Kenawy et al., 2015). In healthy subjects, the leakage of proteins, including complement proteins, to renal tubules is minimal; however, in COVID-19 patients, proteinuria is a common clinical finding (Cheng et al., 2020; Nadim et al., 2020). In fact, complement system activation and deposition of the complement component, C5b-9, in the renal tubules of COVID-19 patients has been reported (Batlle et al., 2020; Benedetti et al., 2020). The complement system promotes tubulointerstitial damage and is a major player in renal injury, especially during acidosis (Kenawy et al., 2015), which can occur during SARS-CoV-2 infection due to hypoxia. In fact, 12% of COVID-19 patients present with acidosis, and the reported percentage among those with AKI is 23% (Chen et al., 2020; Mohamed et al., 2020; Nadim et al., 2020).

The complement system is tightly cross-linked with the coagulation system (Kenawy et al., 2015; Figure 3). Coagulation disorders are commonly observed in severely ill patients with COVID-19, and disseminated intravascular coagulation, ischemic limbs, strokes, and venous thromboembolism have been consistently reported (Liao et al., 2020; Mohamed et al., 2020). Nevertheless, thrombocytopenia, prolonged prothrombin time and higher D-dimer levels have also been observed and associated with death in COVID-19 patients (Liao et al., 2020). Notably, increased coagulation factors are correlated with decreased renal function in subjects without cardiovascular disease or chronic kidney disease (CKD; Dekkers et al., 2018).

Lung involvement in COVID-19 can result in hypoxia (Figure 3). The kidneys are particularly sensitive to changes in oxygen delivery. Persistence of renal hypoxia leads to the activation of intrarenal cellular mechanisms involved in renal fibrosis and vasoconstriction, which in turn enhances renal hypoxia (Haase, 2013; Fu et al., 2016). This cycle contributes to the development of AKI and the progression to CKD (Fu et al., 2016).

Hypotension can be an outcome of SARS-CoV-2 infection and can be associated with hemodynamic instability, shock, or sepsis (Shetty et al., 2020; Figure 3). In addition, orotracheal intubation presents a potential risk of hypotension (Smischney et al., 2016). In a recent report, most COVID-19 patients admitted to the ICU had preserved hemodynamics, unless heart failure, sepsis, or thrombotic events were associated (Corrêa et al., 2020; Hanidziar and Bittner, 2020). In contrast, a case series in the Seattle region reported that the most common cause of ICU admission was hypoxemia or hypotension, and the mortality rate among these patients was extremely high (Bhatraju et al., 2020). The severity and duration of hypotension are closely associated with the risk of AKI development in ICU patients (Lehman et al., 2010).

In conclusion, COVID-19 has a triad of respiratory, inflammatory, and coagulation symptoms that are frequently present in severe ill patients, and that indirectly can promote AKI.

### SARS-CoV-2 Direct Effects on Kidneys: Imbalance of Intrarenal RAS and Disturbance of Kidney Homeostasis

In the kidneys, ACE2 is expressed in podocytes, mesangial cells, parietal epithelium of Bowman’s capsule, brush border proximal cells, and collecting duct cells (Hamming et al., 2008; Aragão et al., 2011). Single-cell RNA sequencing analysis of different kidney cells enabled the identification of a relatively high co-expression of ACE2 and TMPRSS2 in podocytes and proximal straight tubule cells (Pan et al., 2020). Furthermore, the kidney is one of the organs with the highest ACE2 expression and activity (Hamming et al., 2004).

Post-mortem analysis of the kidneys of COVID-19 patients revealed the accumulation of SARS-CoV-2 antigens in the renal epithelial tubules, suggesting a direct infection of the kidneys by the virus (Diao et al., 2020).

Podocytes and proximal straight tubule cells are strong candidates for SARS-CoV-2 host cells in the kidneys, as they participate actively in urine filtration, excretion, and reabsorption. In fact, the virus was detected in urine samples of patients with severe COVID-19 (Diao et al., 2020). In addition, podocytes are extremely sensitive to bacterial and viral infections and podocyte injury leads to proteinuria, a common laboratory finding in COVID-19 patients even upon admission (Cheng et al., 2020; Pan et al., 2020). Kidney autopsy findings in patients with SARS-CoV-2 confirmed the presence of viral particles in the podocytes accompanied by morphological alterations, foot process effacement, vacuolation, and detachment from the glomerular basement membrane (Su et al., 2020).

Considering the direct infection of the kidneys with SARS-CoV-2, ACE2 depletion may be an important factor driving kidney injury. This can result in an imbalance of intrarenal ACE2/Ang 1–7/Mas and ACE/Ang II/AT1 arms of RAS. Additionally, ACE2 depletion may impact the KKS, downregulate BK/B2, and exacerbate desArg^9^BK/B1 actions. These components are involved in renal hemodynamic homeostasis and the molecular mechanisms involved in kidney diseases, including vasoconstriction, oxidative stress, inflammation, and fibrosis (Figure 4). A recent study reported the super activation of RAS in patients with COVID-19 and AKI; the levels of renin and aldosterone were increased and correlated with reduced sodium excretion (Dudoignon et al., 2020). The presence of AKI contributed to a 10-fold increase in mortality in this study (Dudoignon et al., 2020). Thus, we discuss the impact of ACE2 depletion on the kidneys.

Angiotensin-converting enzyme 2 can directly antagonize ACE/Ang II/AT1 actions by cleavage of Ang II with subsequent formation of Ang 1–7. In addition, ACE2 can modulate ACE/Ang II/AT1 antagonism downstream through Ang 1–7, which can downregulate AT1 in vascular smooth cells, inhibit ACE in internal mammalian arteries, activate AT2, and promote vasodilatation (Roks et al., 1999; Clark et al., 2001; Figure 4). This counterregulatory effect is important because super activation of the ACE/Ang II/AT1 axis is related to the deleterious effects within the kidneys (Yang and Xu, 2017).

Ang II has well-documented vasoconstrictor, anti-natriuretic, and anti-diuretic effects (Simóes-e-Silva et al., 1997; Kanaide et al., 2003; Zaika et al., 2013; Wu et al., 2020). Besides, intrarenal Ang II is involved in oxidative stress, inflammation, and fibrosis (Figure 4). Ang II promotes the activity of the transmembrane nicotinamide adenine dinucleotide phosphate (NADPH) oxidase (NOX), which is mainly responsible for the generation of reactive oxygen species (ROS), especially superoxide anion and hydrogen peroxide in the kidneys (Rubattu et al., 2013). NOX-induced oxidative stress contributes to nitric oxide (NO) depletion, imbalance in eicosanoids synthesis, and to a proinflammatory and profibrotic state (Rubattu et al., 2013).

Also, Ang II can mediate profibrotic actions by direct activation of the transcription and synthesis of TGF-β, particularly TGF-β1. Ang II has also been proposed to upregulate the TGF-β receptor (Rüster and Wolf, 2011). TGF-β1 is a profibrotic and anti-inflammatory cytokine that enhances fibronectin and collagen type I mRNA expressions, posteriorly promoting extracellular matrix synthesis and deposition in the interstitial space (Rüster and Wolf, 2011; Macconi et al., 2014). Furthermore, Ang II induces expression of other profibrotic factors, including endothelin-1, plasminogen activator inhibitor-1, matrix metalloproteinase-2, and its tissue inhibitor (Rüster and Wolf, 2011).

In contrast, the peptide formed by the cleavage of Ang II by ACE2, Ang 1–7, promotes counterregulatory action at the intrarenal level (Figure 4). Ang 1–7 mediates vasodilation indirectly by stimulating the actions of BK on B2 and promotes the release of prostacyclin and NO (Schindler et al., 2007; Schinzari et al., 2018). There are controversial reports on the effects of Ang 1–7 on water balance. A diuretic and natriuretic effect has been demonstrated in several animal models and *in vitro* studies (Pinheiro and Simões E Silva, 2012). In contrast, in water-loaded animals, the anti-natriuretic and anti-diuretic effects of Ang 1–7 have been reported (Pinheiro and Simões E Silva, 2012).

Ang 1–7 has protective effects in the kidneys, including antioxidant, anti-inflammatory, and anti-fibrotic effects (Figure 4). These effects of Ang 1–7 are partially due to the inhibition of NFκB signaling and reduction of the levels of chemokines and cytokines, such as MCP-1, TNF-α, IL-1β, ICAM-1, and VCAM-1 (Khajah et al., 2016; Choi et al., 2020). Ang 1–7 interferes with TGF-β1 signaling through Smad2/3 and Smad4, a mechanism that leads to an enhanced synthesis of collagen type I, fibronectin, and α-SMA (Macconi et al., 2014; Choi et al., 2020). Ang 1–7 may also exert antioxidant effects through the stimulation of NO. At physiological concentrations, NO abates oxidative stress by neutralizing some species of ROS. In addition, NO can protect cells from death induced by ROS, such as hydrogen peroxide, alkyl hydroperoxides, and xanthine oxide (Wink et al., 2001).

Not all ACE2 biological functions are attributed to the antagonism of ACE/Ang II/AT1 axis, as previously mentioned. ACE2 can modulate BK downstream, stimulating its effects through B2 and increasing BK half-life by inhibiting the ACE/Ang II/AT1 axis (Cyr et al., 2001; Schindler et al., 2007; Schinzari et al., 2018). Furthermore, ACE2 inactivates the B1 agonist, desArg^9^BK, downregulating its actions (Cyr et al., 2001). Thus, ACE2 is an important regulator of KKS.

Binding of BK to B2 rapidly stimulates the activity of endothelial nitric oxide synthase and prostacyclin synthesis to enhance the release of NO, prostacyclin, and endothelial-derived hyperpolarizing factor, which culminates in a potent and rapid vasodilator response (Figure 4; Hornig and Drexler, 1997; Kakoki and Smithies, 2009). Moreover, intrarenal BK effects on renal hemodynamics are mediated by B2 activation and include an augmented renal blood flow, increased GFR, and diuresis (Zhang et al., 2018). Additionally, BK may modulate oxidative stress and senescence; as impairment of KKS is associated with oxidative damage and mitochondrial dysfunction (Kakoki and Smithies, 2009). BK plays an important role in the inflammatory response, mediating vasodilation and increasing vascular permeability. BK binding to B2 promotes IL-6 expression (Golias et al., 2007).

Under physiological conditions, most of the KKS actions are mediated by B2 activation and the B1 is induced in inflammatory processes (Klein et al., 2010). Infusion of low concentrations of desArg^9^BK in anesthetized rats decreased renal blood flow and GFR, which was associated with increased renal vascular resistance and opposite effects to those of BK (Schanstra et al., 2000; Zhang et al., 2018). B1 activation is associated with enhanced transcription of NFκB, which has a positive feedback on B1 expression and promotes the release of cytokines and chemokines, ultimately resulting in the accumulation of leukocytes (Klein et al., 2010).

In conclusion, ACE2 downstream regulates RAS, KKS, prostaglandins, and NO, all involved in the renal pathophysiology. Thus, ACE2 depletion at the renal level has a significant impact on kidney function and on the molecular mechanisms involved in kidney injury.

## Conclusion

Currently, AKI is recognized as a frequent complication of COVID-19. Considering the poor prognostics associated with presence of AKI in COVID-19 patients, elucidating the mechanisms involved in SARS-CoV-2-induced kidney injury is fundamental for developing strategies to better manage these patients. Furthermore, there is no available information on the impact of COVID-19 on long-term kidney function. The possibility of AKI evolving to CKD is a concern as patients with AKI are more prone to develop CKD and end-stage renal disease; severe conditions linked to high personal, societal, and economic burdens (Chawla et al., 2017).

Currently, there is insufficient information regarding AKI recovery in discharged COVID-19 patients. Most patients can recover from AKI stage 1, while those who evolve to stages 2 and 3 have a high mortality rate. The COVID-19-related AKI seems to contribute to a faster decline in GFR, higher rate of KRT requirement, and slower complete kidney function recovery (Chan et al., 2020; Xiao et al., 2020; Nugent et al., 2021). Such findings highlight the importance of monitoring kidney function in COVID-19 survivors who presented AKI or kidney abnormalities. Clinical interventions during the time frame between AKI and possible establishment of CKD are essential to alter the course of the disease (Chawla et al., 2017).

The triad of symptoms of COVID-19, namely inflammation, impaired immune response, and coagulation disorders, can indirectly affect kidney homeostasis and cause kidney injury. Pulmonary impairment with hypoxemia and the involvement of the heart also induce renal damage. The kidneys are a potential target for direct SARS-CoV-2 infection. In this scenario, besides the damage caused by viral infection and replication, the depletion of ACE2 can be an important mechanism leading to the imbalance of RAS and KKS, contributing to a cascade of intrarenal cellular mechanisms involved in kidney injury. The impairment of ACE2 and consequently RAS and KKS, may persist after COVID-19 infection, compromising the long-term kidney function. Considering this, an extensive study of the modulation of intrarenal RAS, especially ACE2, in the context of COVID-19 may hold a key to establishing therapies to manage COVID-19-induced kidney injury.

## Author Contributions

NA and LG selected the relevant publications, wrote, and generated the figures for this manuscript. HT and JO contributed to the conception and revision of the article. DC was responsible for the design of this manuscript and critical assessment of the content based on her expertise. All authors contributed to the article and approved the submitted version.

## Conflict of Interest

The authors declare that the research was conducted in the absence of any commercial or financial relationships that could be construed as a potential conflict of interest.

## Publisher’s Note

All claims expressed in this article are solely those of the authors and do not necessarily represent those of their affiliated organizations, or those of the publisher, the editors and the reviewers. Any product that may be evaluated in this article, or claim that may be made by its manufacturer, is not guaranteed or endorsed by the publisher.

## Abbreviations

AKI: Acute kidney injury
ARDS: ACUTE respiratory distress syndrome
Ang: Angiotensin
ACE: Angiotensin-converting enzyme
ACE2: Angiotensin-converting enzyme 2
ARB: Angiotensin II receptor blocker
AT1: Angiotensin II receptor of type 1
AT2: Angiotensin II receptor of type 2
B1: Kinin receptor type 1
B2: Kinin receptor type 2
BK: Bradykinin
CKD: Chronic kidney disease
COPD: Chronic obstructive pulmonary disease
COVID-19: Coronavirus disease 2019
desArg^9^BK: desArg^9^bradykinin
ENaC: Epithelial Na^+^ channel
GFR: Glomerular filtration rate
ICU: Intensive care unit
IL: Interleukin
KKS: Kallikrein-kinin system
KRT: Kidney replacement therapy
LPS: Lipopolysaccharides
MERS-CoV: Middle East respiratory syndrome coronavirus
MAPK: Mitogen activated protein kinases
MCP-1: Monocyte chemoattractant protein 1
NADPH: Nicotinamide adenine dinucleotide phosphate
NCC: NaCl cotransporter
NO: Nitric oxide
NOX: NADPH oxidase
NFκB: Nuclear factor kappa B
PM2: Pneumocytes type 2
ROS: Reactive oxygen species
RBD: Receptor binding domain
RAS: Renin-angiotensin system
S: Spike protein
SARS-CoV: Severe acute respiratory syndrome coronavirus
SARS-CoV-2: Severe acute respiratory syndrome coronavirus 2
TMPRSS2: Transmembrane serine protease 2
TNF: Tumor necrosis factor
